# Biotransformation of gluten-free composite flour mediated by probiotics *via* solid-state fermentation process conducted under different moisture contents

**DOI:** 10.3389/fnut.2023.910537

**Published:** 2023-02-15

**Authors:** Kareem Adebayo Koyum, Hooi Ling Foo, Norhayati Ramli, Teck Chwen Loh

**Affiliations:** ^1^Department of Bioprocess Technology, Faculty of Biotechnology and Biomolecular Sciences, Universiti Putra Malaysia, Serdang, Malaysia; ^2^Research Laboratory of Probiotics and Cancer Therapeutics, Institute of Bioscience, Universiti Putra Malaysia, Serdang, Malaysia; ^3^Laboratory of Biopolymer and Derivatives, Institute of Tropical Forestry and Forest Products (INTROP), Universiti Putra Malaysia, Serdang, Malaysia; ^4^Department of Animal Science, Faculty of Agriculture, Universiti Putra Malaysia, Serdang, Malaysia

**Keywords:** composite flour, rice, sorghum, soybean, probiotics, biotransformation, solid-state fermentation, nutritive quality

## Abstract

Staple foods produced from composite flour are considered feasible to alleviate protein-energy malnutrition (PEM). However, one of the major limitations of composite flour is poor protein digestibility. The biotransformation process mediated by probiotics *via* solid-state fermentation (SSF) holds a promising potential to address the poor protein digestibility in composite flour. Yet, there is no report established in this regard to the best of our knowledge. Therefore, 4 strains of *Lactiplantibacillus plantarum* and *Pediococcus pentosaceus* UP2 isolated from Malaysian foods that were previously reported to produce versatile extracellular hydrolytic enzymes were employed to biotransform gluten-free composite flour derived from rice, sorghum, and soybean. The SSF process was performed under 30–60% (v/w) moisture content for 7 days, where samples were withdrawn at 24 h intervals for various analyses such as pH, total titratable acidity (TTA), extracellular protease activity, soluble protein concentration, crude protein content, and *in vitro* protein digestibility. The pH of the biotransformed composite flour showed a significant reduction from the initial range of pH 5.98–6.67 to the final pH of 4.36–3.65, corresponding to the increase in the percentage of TTA in the range of 0.28–0.47% to 1.07–1.65% from days 0 to 4 and remained stable till day 7 of the SSF process. The probiotics strains exhibited high extracellular proteolytic activity (0.63–1.35 U/mg to 4.21–5.13 U/mg) from days 0 to 7. In addition, the treated composite flour soluble protein increased significantly (*p* ≤ 0.05) (0.58–0.60 mg/mL to 0.72–0.79 mg/mL) from days 0 to 7, crude protein content (12.00–12.18% to 13.04–14.39%) and protein digestibility (70.05–70.72% to 78.46–79.95%) from days 0 to 4 of SSF. The results of biotransformation of 50% (v/w) moisture content were mostly comparable to 60% (v/w) moisture content, implying 50% (v/w) moisture content was the most suitable moisture content for the effective biotransformation of gluten-free composite flour mediated by probiotics *via* SSF since flour quality is better at lower moisture content. As for the overall performance, *L. plantarum* RS5 was ranked the best strain, attributed to the general improvement in the physicochemical properties of composite flour.

## Introduction

Protein-energy malnutrition (PEM) remains a major health burden in developing countries. One of the key contributing factors to PEM is the high consumption of low-quality diets (1, 2). Food grains such as cereals and legumes provide the primary dietary source of protein, fat, vitamins, and minerals for people in developing countries (3). According to reports, nearly 3.5 billion people eat rice, with the highest consumption rate in Asia (4), while approximately 300 million people in Africa rely on sorghum-based staples (5). Similarly, soybean is a popular legume that serves as the main source of protein in Asia and other parts of the world (6). Although rice, sorghum, and soybean are cheap and readily accessible for vulnerable populations, over-reliance on single foods (a monotonous diet) can result in nutritional deficiencies (7). Hence, composting cereals and legumes to produce better nutritious food is considered an inexpensive method of addressing the PEM challenge (8). Rice, sorghum, and soybean composite blends are gluten-free and are available in developing countries to replace wheat-based composite flour (9–11). However, despite the numerous advantages of composite flour (3), the high amount of anti-nutritional factors is typical of food derived from plant origin, which usually impedes nutrient bioavailability. Cereals and legumes contain anti-nutritional factors such as phytate, tannin, oxalate, and protease inhibitors that interfere with protein digestibility and mineral bioavailability (3, 8, 12).

Protein quality relates to both protein digestibility and the bioavailability of amino acids. Although research efforts on composite flour over the years have focused on improving the protein content and lysine limitation in cereal-based food products (3, 8, 13), limited studies have been conducted to address the nutritional limitations of composite flour (14). The purpose of food processing is to improve food quality and organoleptic characteristics, whereas the conventional food fermentation technique has been well recognized for its potential to solve malnutrition issues in countries with limited infrastructure (12, 13). Similarly, the adoption of a cost-effective and eco-friendly solid-state fermentation (SSF) technique as a strategy to transform agricultural biomass into useful bioproducts has become a new paradigm shift in biotechnological research (15, 16). The biotransformation process mediated by enzymes or intact microorganism cells usually engages the SSF technique to transform solid substrates into various valuable products (17). An array of microorganisms have been reported to produce several industrial enzymes, such as protease, amylase, pectinase, lipase, and phytase, to improve the nutritive quality of staple foods (15). However, despite a lack of free water in SSF that is mainly suitable for fungal growth, probiotics lactic acid bacteria (LAB), which are considered safe for human consumption, have been recently applied as a biotransformation agent to improve human food and animal feed quality (6, 16, 18–20).

Previously, the effects of various modes of fermentation involving single food grains (such as rice, sorghum, and soybean) and co-fermentation of sorghum and soybean to develop composite flour food products have been documented (10, 18, 21–27). However, there is no report on the biotransformation process mediated by LAB *via* the SSF technique on composite flour. Strains of probiotics LAB, such as *Lactiplantibacillus plantarum* RG14, *Lactiplantibacillus plantarum* RI11, *Lactiplantibacillus plantarum* RS5, *Lactiplantibacillus plantarum* UL4, and *Pediococcus pentosaceus* UP2, isolated from Malaysian foods were recently reported for their versatile capabilities of producing extracellular hydrolytic enzymes under SSF conditions and exhibited high amino-acids production capabilities (16, 28, 29). However, previous applications of these probiotics LAB and their post-biotic metabolites have mainly focused on improving feed quality and the impacts on the growth and health of livestock (16, 30–33). Therefore, this study aimed to evaluate the biotransformation effects of the five selected probiotics *L. plantarum* and *P. pentosaceus* UP2 strains on the pH, total titratable acidity (TTA), and protein digestibility of gluten-free composite flour derived from rice, sorghum, and soybean *via* SSF process conducted under different moisture contents.

## Materials and methods

Rice (Cap kapal layar parboiled rice), sorghum (Lohas organic sorghum white variety), and soybean (China OEM dried yellow seeds) grains were purchased from a local supermarket in Seri Kembangan, Selangor, Malaysia. The probiotics LAB *L. plantarum* RG14 (GU138145), *L. plantarum* RI11 (GU138147), *L. plantarum* UL4 (GU138143), *L. plantarum* RS5 (GU138148), and *P. pentosaceus* UP2 were obtained from the Industrial Biotechnology Laboratory, Department of Bioprocess Technology, Faculty of Biotechnology and Biomolecular Sciences, Universiti Putra Malaysia (UPM). The stock cultures were maintained in de Man, Rogosa, and Sharpe (MRS) medium supplemented with 20% (v/v) glycerol and stored at −20°C upon collection. All reagents used in this experiment were of analytical grade and purchased from Sigma-Aldrich Corporation.

### Preparation of rice, sorghum, and soybean flour

Rice, sorghum, and soybean grains were milled separately to 0.5 mm flour and dried overnight to remove excess moisture (16, 34).

### Formulation and preparation of composite flour from rice, sorghum, and soybean

Rice, sorghum, and soybean flour were weighed in the ratio of 45:45:10 (4, 5, 9–11, 35) and uniformly mixed for 30 min using a food processor (Kenwood, Hampshire Britain).

### Maintenance of LAB culture

The reviving of the stock culture was carried out following the procedure of Foo et al. (30). In brief, 1% (v/v) of glycerol stock culture was introduced into 10 ml of MRS broth and incubated for 48 h at 30°C. Thereafter, 1% (v/v) of the 48-h culture was transferred into another 10 ml of MRS broth and incubated for 24 h at 30°C. A loopful of the 24 h culture was streaked onto MRS agar and incubated for 48 h at 30°C. A single and distinct colony was picked and subcultured into a freshly prepared 10-ml MRS broth and incubated for 48 h. Finally, 1% (v/v) of the 48 h culture was transferred to 10 ml MRS broth and incubated for 24 h at 30°C to obtain a ready-to-use (RTU) culture.

### Preparation of inoculum

The cell biomass of RTU culture was collected by centrifugation at 10,000 × *g* for 15 min (Hitachi Himac CR22611 High-Speed Refrigerated Centrifuge, Hitachi Koki Co Ltd., Tokyo, Japan) and washed with sterile 0.85% (w/v) NaCl solution (Merck, Darmstadt, Germany). The cell population of RTU suspension was adjusted to approximately 10^9^ CFU/ml using sterile 0.85% (w/v) NaCl solution [Absorbance (Abs) of 1.00 at 600 nm wavelength (Cary 50 Probe UV-visible spectrophotometer, Agilent Technologies, Santa Clara, CA, USA) (16)].

### Solid-state fermentation of composite flour

Fermentation of composite flour was carried out according to the method of Alshelmani et al. (36). The amount of 1 kg composite flour was sprayed with 10% (w/v) of 10^9^ CFU/ml of LAB culture and mixed thoroughly. While mixing, deionized water was added to the blend and adjusted to obtain 30% [0.3:1 (v/w)], 40% (0.4:1 (v/w)], 50% (0.5:1 (v/w)], and 60% (0.6:1 (v/w)] total moisture content (MC) of blend:water, respectively. There were two control samples in this study, whereby the positive controls were prepared by substituting the volume of LAB culture with deionized water water-treated control (WTC), whereas the negative control was prepared with the raw composite flour with no addition of deionized water and culture, which served as the non-treated control (NTC). After mixing, 150 g of the composite flour mixture was sealed in an airtight plastic bag and allowed to ferment at room temperature for 7 days. The samples were withdrawn at 24-h intervals and kept at −20°C for various analyses.

### Physicochemical and microbiological analyses

#### Viable cell count

For each sample, 1 g of the biotransformed composite flour mixture was mixed with 9 ml of sterile 0.85% (w/v) NaCl solution, and 1 ml of the suspension mixture was serially diluted with a 10-fold dilution factor. The diluted sample was evaluated for viable cell count using MRS and Eosin Methylene Blue (EMB) agar (Merck, Darmstadt, Germany) to determine the LAB and *Enterobacteriaceae* growth, respectively. The bacterial growth was expressed as log_10_ colony forming unit (CFU)/g (16). All analyses were performed in triplicates.

#### pH and total titratable acidity (TTA)

The 10 g of biotransformed composite flour was mixed with 90 ml of deionized water in an Erlenmeyer flask prior to pH determination using a pH meter (Mettler Toledo, Malaysia). Subsequent determination of TTA was carried out by titrating the solution against 0.1 M of NaOH using phenolphthalein as an indicator. The percentage of TTA was calculated with the volume of NaOH used (37) for the titration. All analyses were performed in triplicates.


$$\%\mathrm{Acid}\left(\text{wt}./\text{vol}\right)=\frac{\text{N}\times V\times \mathrm{Eq}.\text{wt}.\mathrm{of}\mathrm{Lactic}\mathrm{acid}}{\text{W×}1000}\text{×}100$$

where *N* = normality of NaOH, *V* = volume of NaOH, and *W* = weight of composite flour.

#### Extraction of crude enzymes from biotransformed composite flour

The 10 g of biotransformed composite flour was mixed with 10 ml of deionized water in a 50-ml Falcon tube, followed by agitation at 130 rpm using an incubator shaker (Innovar 42 Incubator Shaker Series, New Brunswick Scientific, Hamburg, Germany) for 30 min at 30°C. The resulting mixture was centrifuged at 12,000 × *g* at 4°C for 20 min (Himac CR 22GII, Hitachi High-Speed Refrigerated Centrifuge, Hitachi Koki Co Ltd., Tokyo, Japan). The clear supernatant was collected and filtered through a cellulose acetate membrane (Sartorius Minisart, 0.22 μm, Göttingen, Germany) and stored at −20°C (16, 36) for the determination of extracellular enzyme activity, as described below.

#### Determination of protease activity extracted from biotransformed composite flour

Protease activity was determined using the method described by Thung (38). A solution of 2.5% (w/v) azocasein (Sigma Aldrich, St. Louis, MO, USA) was prepared as the substrate; the biotransformed composite flour extract was used as a crude enzyme, while the protease activity was determined at pH 5 using 0.1-M sodium acetate (Merck, Darmstadt, Germany) buffer solution. In brief, a mixture of the substrate and biotransformed composite flour extract was incubated at 37°C water bath for 30 min. Next, 750 μl of 10% (w/v) trichloroacetic acid (TCA) (Merck, Darmstadt, Germany) was added and allowed to equilibrate at room temperature for 30 min to precipitate the azocasein. Then, the assay mixture was centrifuged at 12,000 × *g*, 4°C for 10 min to remove the precipitates. Thereafter, 600 μl of the supernatant was mixed with 600 μl of 0.1 M of NaOH (Merck, Darmstadt, Germany) and allowed to equilibrate at room temperature for 15 min. The Abs was measured at 450 nm. One unit of protease activity (U/mg) was expressed as the enzyme capable of hydrolyzing azocasein to produce 0.001 in Abs change per min of reaction time per mg of enzyme protein under assay conditions.

#### Determination of soluble protein extracted from biotransformed composite flour

The biotransformed composite flour extract was used to determine the soluble protein concentration. In brief, 0.5 ml of the diluted extract was mixed with 0.5 ml of Bradford reagents (Sigma Aldrich, St. Louis, MO, USA) and incubated at 4°C for 5 min. The Abs was measured at 595 nm, and bovine serum albumin (BSA) (Sigma Aldrich, St. Louis, MO, USA) was used as the reference (39). Soluble protein concentration was expressed as mg/ml of the sample.

#### Determination of protein content of biotransformed composite flour

The protein content of the biotransformed composite flour was evaluated, as described in the standard method 920.87 of AOAC (37). In brief, 0.5 g of dried raw or biotransformed composite flour was digested using 12 ml of concentrated H_2_SO_4_ (Sigma Aldrich, St. Louis, MO, USA) and 1 tablet of catalyst in a Kjeldahl flask by heating to obtain a clear straw color solution. The digested sample was subjected to a distillation and titration process using an automatic Kjeldahl nitrogen protein analyzer, and the protein content was expressed as the percentage of the protein content.

#### Determination of *in vitro* protein digestibility of biotransformed composite flour

The modified method of Akeson and Stahmann (40), as described by Byanju et al. (23), was used to determine the *in vitro* protein digestibility. In brief, 0.25 g of dried raw or biotransformed composite flour and 250 μl of deionized water (blank) were first treated with gastric digestion using 0.1 M of HCl containing 1.5 mg/ml of pepsin (Sigma Aldrich, St. Louis, MO, USA) and incubated at 37°C for 3 h. The mixture was then neutralized with 0.5 M of NaOH prior to pancreatic digestion using 10 ml of 0.2-M phosphate buffer solution containing 10 mg of pancreatin (Sigma Aldrich, St. Louis, MO, USA) and 1 ml of 0.005 M sodium azide (Merck, Darmstadt, Germany) to prevent microbial growth. Then, the sample mixture was incubated at 37°C for 24 h. Next, 1 ml of 10% (w/v) TCA was added to the mixture and centrifuged at 500 × *g* for 20 min. The supernatant was collected, and the total protein content was determined using the Bradford assay method (39). The *in vitro* protein digestibility was calculated with the formula below:


$$\%\text{ Digestible Protein}=\left({\text{P}}_{\text{s}}–{\text{P}}_{\text{b}}\right){\text{/P}}_{\text{s}}\times \text{1}00,$$

where P_*s*_ and P_*b*_ represent the protein content in the supernatant and the blank, respectively.

#### Statistical analysis

The mean of each experiment conducted in triplicates was subjected to analysis of variance (ANOVA), and the Tukey’s *post-hoc* test was carried out at a significance level of 0.05 (GraphPad Prism Software, Version 9.2.0, San Diego, CA, USA). The linear regression for the interaction between the moisture content and the biotransformation period (days) was performed for all the analyzed variables using GraphPad Prism Software (Version 9.2.0, San Diego, CA, USA).

## Results

### Effect of moisture contents on the cell viability of probiotics

Table 1 presents the effect of MCs on the cell viability of the selected probiotic strains. The initial population of the selected probiotic strains varied between 7.08 and 8.38 log_10_ CFU/g across 30–60% (v/w) MC on day 0, while the initial LAB population recorded in WTC was between 3.55 and 4.67 log_10_ CFU/g. Subsequently, on day 1 of the SSF process, an increase in the viable cell count of the selected probiotics strains was observed across 30–60% (v/w) MC, while the LAB population in the WTC remained the same. In this period, the highest viable cell count for the selected probiotic strains was recorded at 60% (v/w) MC by the RS5 strain (10.12 log_10_ CFU/g) followed by RG14 (10.06 log_10_ CFU/g), UL4 (9.55 log_10_ CFU/g), RI11 (9.47 log_10_ CFU/g), and UP2 (9.35 log_10_ CFU/g), whereas the lowest increase in viable cell count was noted at 30% (v/w) MC (RG14, 9.17 log_10_ CFU/g; RS5, 8.89 log_10_ CFU/g; RI11, 8.67 log_10_ CFU/g; UL4, 8.65 log_10_ CFU/g and UP2 8.55 log_10_ CFU/g). Furthermore, the population of the selected probiotics maintained stability across 30–60% (v/w) MC from days 1 to 5, respectively, except for RG14, RS5, and UP2 strains that recorded a slight drop in the population at 50–60% on day 2 and thereafter maintained a stable growth until day 4. On the other hand, the LAB population in the WTC consistently increase from days 2 to 6 across 30–60% (v/w) MC reaching the highest viable count of 7.21 log_10_ CFU/g at 60% (v/w) MC and the lowest viable count of 5.34 log_10_ CFU/g at 30% (v/w) MC on day 5, respectively. Finally, a general decrease in the population of the selected probiotic strains and LAB in the WTC was recorded from days 5 to 7.

**TABLE 1:** Effect of moisture contents on the cell viability of probiotic LAB strains during solid-state fermentation of composite flour for 7 days.

Viable cell count (Log_10_ CFU/g)						
Days	WTC	RI11	RG14	RS5	UL4	UP2
**(a) 30% (v/w) moisture content**						
0	3.55 ± 0.07^aA^	7.61 ± 0.03^bcA^	7.05 ± 0.02^dA^	7.74 ± 0.03^bceA^	7.87 ± 0.02^bdA^	7.06 ± 0.04^bcA^
1	4.16 ± 0.01^aA^	8.67 ± 0.02^bB^	9.17 ± 0.04^beB^	8.89 ± 0.06^bcfB^	8.65 ± 0.04^bdB^	8.55 ± 0.04^bAB^
2	4.80 ± 0.06^bB^	8.42 ± 0.01^aC^	8.71 ± 0.05^acfB^	8.75 ± 0.09^cfgBD^	8.60 ± 0.02^fB^	8.53 ± 0.04^afBC^
3	5.05 ± 0.08^aB^	8.45 ± 0.01^bBC^	8.43 ± 0.01^bBC^	8.76 ± 0.09^bceBC^	8.55 ± 0.01^cB^	8.55 ± 0.03^bcBD^
4	5.25 ± 0.01^aB^	8.44 ± 0.01^bBC^	8.33 ± 0.07^bcBC^	8.41 ± 0.03^bBD^	8.38 ± 0.05^bBC^	8.30 ± 0.04^bAC^
5	5.34 ± 0.01^aC^	8.07 ± 0.03^beAB^	8.50 ± 0.02^cBC^	8.40 ± 0.02^dcBD^	7.93 ± 0.02^eC^	8.30 ± 0.02^bdB–D^
6	5.18 ± 0.04^aD^	7.30 ± 0.27^a–lABC^	8.39 ± 0.05^bBC^	8.16 ± 0.07^bchACD^	7.42 ± 0.01^dcfA^	7.87 ± 0.05^ecAD^
7	4.92 ± 0.03^aD^	7.28 ± 0.16^b–fAC^	8.37 ± 0.06^dC^	7.80 ± 0.03^eC^	7.33 ± 0.01^fA^	7.87 ± 0.05d^eAD^
**(b) 40% (v/w) moisture content**						
0	3.71 ± 0.07^aB^	7.74 ± 0.02^bC^	7.19 ± 0.02^cdeD^	7.82 ± 0.03^bcdA^	7.41 ± 0.07^bcdA^	7.23 ± 0.07^bcdCD^
1	5.39 ± 0.05^aC^	9.19 ± 0.01^ceA^	9.64 ± 0.05^cAB^	9.22 ± 0.07^bcB^	9.06 ± 0.03^c–eB^	9.01 ± 0.04^c–eBC^
2	5.30 ± 0.04^bA^	8.99 ± 0.03^cA^	8.82 ± 0.01^cfBC^	9.08 ± 0.04^cghB^	9.10 ± 0.01^gB^	9.07 ± 0.03^giBC^
3	5.34 ± 0.02^aA^	9.06 ± 0.04^ceA^	8.91 ± 0.06^bceC^	9.02 ± 0.05^cfB^	9.11 ± 0.03^ciB^	8.89 ± 0.02^ckBC^
4	5.11 ± 0.03^aAC^	8.90 ± 0.06^bdfA^	8.87 ± 0.04^bdeBC^	9.11 ± 0.04^defB^	8.98 ± 0.01^cfB^	8.90 ± 0.03^cfgB^
5	5.36 ± 0.03^aA^	8.17 ± 0.03^beB^	8.86 ± 0.04^ckBC^	8.87 ± 0.00^fkB^	8.03 ± 0.07^bceflA^	8.93 ± 0.04^jAC^
6	5.87 ± 0.02^bfD^	7.45 ± 0.13^bfBC^	8.81 ± 0.02^bcfBC^	8.10 ± 0.05^bfA^	7.66 ± 0.05^gcA^	8.55 ± 0.03^bfC^
7	5.37 ± 0.02^cA^	7.35 ± 0.09^befC^	8.80 ± 0.05^bdB^	8.08 ± 0.04^bdeA^	7.40 ± 0.01^bdA^	8.20 ± 0.03^bdfD^
**(c) 50% (v/w) moisture content**						
0	4.14 ± 0.08^aA^	7.81 ± 0.02^bcB^	7.60 ± 0.05^cdC^	8.27 ± 0.00^beA^	7.71 ± 0.02^bcA^	7.64 ± 0.02^bdeA^
1	4.39 ± 0.05^aA^	9.35 ± 0.01^cA^	9.60 ± 0.03^cB^	9.80 ± 0.02^fgB^	9.30 ± 0.01^cB^	9.28 ± 0.02^cBD^
2	6.19 ± 0.01^eB^	9.39 ± 0.01^dC^	9.31 ± 0.03^dghA^	9.27 ± 0.03^dhC^	9.43 ± 0.03^dhB^	9.42 ± 0.01^dhB^
3	6.21 ± 0.05^gBC^	9.43 ± 0.03^eC^	9.26 ± 0.03^efiA^	9.44 ± 0.01^djC^	9.42 ± 0.01^efB^	9.42 ± 0.00^efiB^
4	6.45 ± 0.00^iC^	9.23 ± 0.02^djkC^	9.25 ± 0.01^dkA^	9.25 ± 0.01^dkC^	9.42 ± 0.04^ejkB^	9.22 ± 0.02^djkC^
5	6.14 ± 0.06^iBC^	8.12 ± 0.02^edB^	9.19 ± 0.04^dkAB^	8.91 ± 0.09^c–ekAC^	8.70 ± 0.03^cfC^	9.11 ± 0.02^kCD^
6	6.02 ± 0.03^fcB^	8.14 ± 0.07^bgB^	8.70 ± 0.03^bhD^	8.34 ± 0.02^bhA^	7.89 ± 0.03^hgA^	8.05 ± 0.02^bhgE^
7	5.41 ± 0.04^cD^	7.99 ± 0.02^bdeB^	8.67 ± 0.04^bdAD^	8.31 ± 0.02^bdeA^	7.46 ± 0.00^bD^	8.21 ± 0.08^bdfAE^
**(d) 60% (v/w) moisture content**						
0	4.67 ± 0.18^aAC^	7.93 ± 0.04^bcfB^	7.39 ± 0.01^bcA^	8.38 ± 0.01^efA^	8.06 ± 0.08^cdfB^	8.14 ± 0.08^befA^
1	4.60 ± 0.07^aA^	9.46 ± 0.01^cC^	10.06 ± 0.00^fgB^	10.12 ± 0.01^gB^	9.54 ± 0.03^cA^	9.47 ± 0.00^cB^
2	6.33 ± 0.02^eBC^	9.47 ± 0.00^hC^	9.44 ± 0.00^dhC^	9.45 ± 0.00^dhC^	9.53 ± 0.03^dhiAC^	9.36 ± 0.01^iC^
3	6.34 ± 0.06^gC^	9.33 ± 0.01^efiC^	9.28 ± 0.01^efiC^	9.44 ± 0.01^efCD^	9.51 ± 0.03^dAC^	9.28 ± 0.07^efijABC^
4	6.38 ± 0.04^iC^	9.38 ± 0.04^fkB^	9.14 ± 0.02^dfkD^	9.23 ± 0.01^dgkD^	9.26 ± 0.03^kC^	9.09 ± 0.08^bfkAC^
5	7.21 ± 0.01^ilD^	8.94 ± 0.06^bcdfA^	8.81 ± 0.02^fkE^	9.01 ± 0.04^bfkDE^	8.87 ± 0.02^fkE^	8.88 ± 0.06^cdfkAC^
6	6.60 ± 0.05^iA^	7.97 ± 0.03^bcB^	8.69 ± 0.07^bcE^	8.74 ± 0.04^cfDE^	8.61 ± 0.04^cfBE^	8.81 ± 0.02^cfAC^
7	6.42 ± 00^gAC^	7.95 ± 0.03^eB^	8.65 ± 0.04^dE^	8.74 ± 0.03^dE^	8.18 ± 0.04^fE^	8.70 ± 0.02^dfA^

Values are mean ± standard error of the mean (SEM). Mean values in the same column having the same superscript (a–l) are not significantly different at *p* ≤ 0.05. Mean values in the same row having the same superscript (A–E) are not significantly different at *p* ≤ 0.05. NTC, non-treated control; NTC, LAB growth 0.00 log_10_ CFU/g; WTC, water-treated control; R111, *L. plantarum* RI11; RG14, *L. plantarum* RG14; RS5, *L. plantarum* RS5; UL4, *L. plantarum* UL4; UP2, *P. pentosaceus* UP2. (a) 30% (v/w) moisture content, (b) 40% (v/w) moisture content, (c) 50% (v/w) moisture content, and (d) 60% (v/w) moisture content. *Enterobacteriaceae* was not observed on EMB agar for NTC, WTC, and probiotic-treated composite flours.

### Effects of moisture contents on the pH and percentage of total titratable acidity (%TTA) of biotransformed composite flour

**FIGURE 1:**
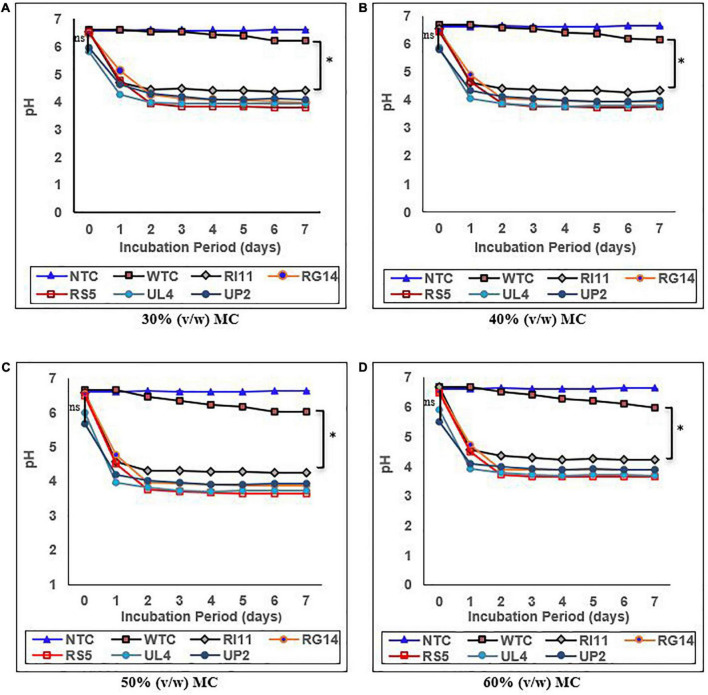
Effect of moisture contents on pH of composite flour during 7 days of solid-state fermentation mediated by probiotic strains. Each value represents mean ± SEM, *n* = 3. NTC, non-treated control; WTC, water-treated control; R111, *L. plantarum* RI11; RG14, *L. plantarum* RG14; RS5, *L. plantarum* RS5; UL4, *L. plantarum* UL4; UP2, *P. pentosaceus* UP2. **(A)** 30% (v/w) moisture content, **(B)** 40% (v/w) moisture content, **(C)** 50% (v/w) moisture content, **(D)** 60% (v/w) moisture content. *Indicate significant difference (p ≤ 0.05). *^ns^*Not significantly different.

**FIGURE 2:**
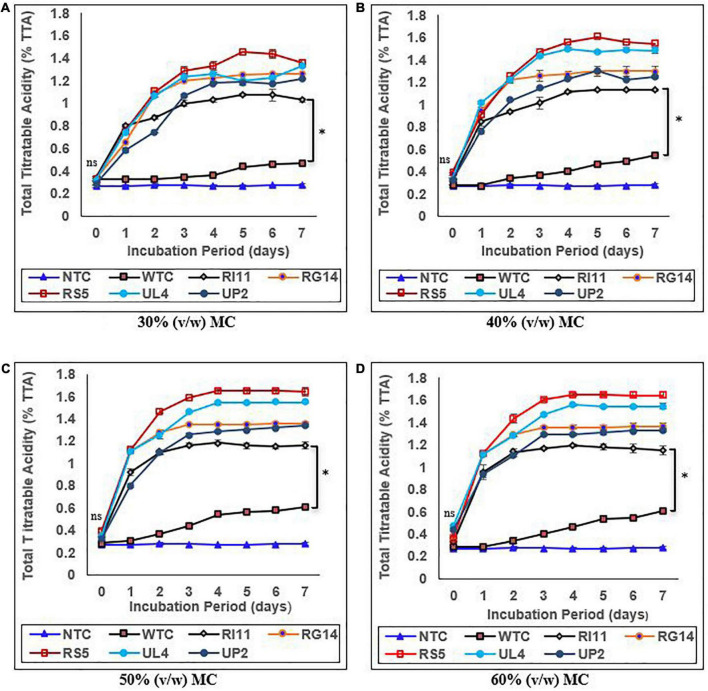
Effect of moisture contents on the percentage of total titratable acidity of composite flour during 7 days of solid-state fermentation mediated by probiotic strains. Each value represents mean ± SEM, *n* = 3. NTC, non-treated control; WTC, water-treated control; R111, *L. plantarum* RI11; RG14, *L. plantarum* RG14; RS5, *L. plantarum* RS5; UL4, *L. plantarum* UL4; UP2, *P. pentosaceus* UP2. **(A)** 30% (v/w) moisture content, **(B)** 40% (v/w) moisture content, **(C)** 50% (v/w) moisture content, **(D)** 60% (v/w) moisture content. *Indicate significant difference (*p* ≤ 0.05). *^ns^*Not significantly different.

The results of the effect of moisture contents on the pH and %TTA are presented in Figures 1, 2, respectively. Moisture content had a significant impact (*p* ≤ 0.05) on the pH and %TTA of the biotransformed composite flour. The highest reduction in pH with a corresponding increase in %TTA was recorded in the probiotic-treated composite flour at 30–60% (v/w) MC, whereas the WTC recorded the least changes. Although no significant difference (*p* ≥ 0.05) in the value of pH and %TTA was obtained for the treated composite, RS5-treated composite flour at 60% (v/w) MC recorded the highest reduction in pH (6.49–3.65) from days 0 to 4, with a corresponding increase in %TTA of 0.36–1.65 followed by UL4 (pH 3.70; %TTA 1.55), RG14 (pH 3.90; %TTA 1.36); UP2 (pH 3.91; %TTA 1.30), and RI11 (4.23; %TTA 1.20) between days 3 and 4 and remained steady till day 7, whereas WTC recorded the lowest reduction in pH from 6.66 to 5.98 and %TTA 0.29–0.61 on day 6. A similar trend in results (*p* ≥ 0.05) with 60% (v/w) MC was noted at 50% (v/w) MC. The least changes in the pH and %TTA of the biotransformed composite flour were observed at 30% (v/w) MC, whereby RS5 strain-treated composite flour maintained the highest pH reduction (6.44–3.75) and %TTA (0.33–1.45) from days 0 to 5, followed by UL4, RG14, UP2, and RI11-treated composite flour from days 0 to 7, respectively.

### Proteolytic activity of biotransformed composite flour extract

**FIGURE 3:**
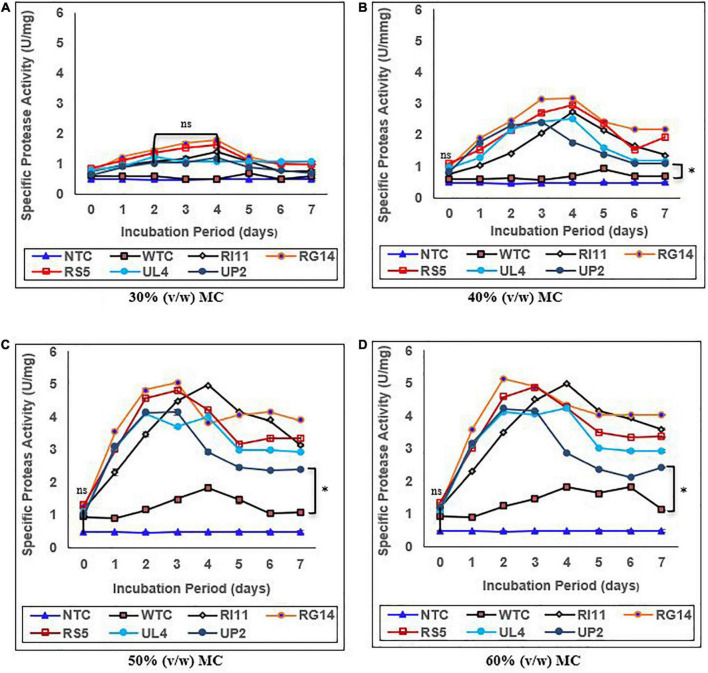
Effect of moisture contents on the specific endoprotease activity of composite flour during 7 days of solid-state fermentation mediated by probiotic strains. Each value represents mean ± SEM, *n* = 3. NTC, non-treated control; WTC, water-treated control; R111, *L. plantarum* RI11; RG14; *L. plantarum* RG14; RS5, *L. plantarum* RS5; UL4, *L. plantarum* UL4; UP2, *P. pentosaceus* UP2. **(A)** 30% (v/w) moisture content, **(B)** 40% (v/w) moisture content, **(C)** 50% (v/w) moisture content, **(D)** 60% (v/w) moisture content. *Indicate significant difference (*p* ≤ 0.05). *^ns^*Not significantly different.

The specific endoprotease activity of WTC and treated composite flour extracts is presented in Figure 3. The specific endoprotease activity of the probiotics-treated composite flour reached its maximum value (*p* ≤ 0.05) between days 2 and 4 across 30–60% (v/w) MC compared to the WTC that showed a slight increase toward the end of the SSF process at 50% and 60% (v/w) MC. The highest endoprotease activity of 5.13 U/mg was demonstrated by RG14-treated composite flour at 60% (v/w) MC, followed by RI11 (4.98 U/mg), RS5 (4.88 U/mg), UL4, UP2, and WTC (1.82 U/mg). In addition, the results obtained had a similar trend to the endoprotease activity recorded at 50% (v/w) MC. Furthermore, the lowest increase in specific endoprotease activity was noted at 30% (v/w) MC by RG14 (1.78 U/mg), RS5 (1.62 U/mg), RI11 (1.39 U/mg), UL4 (1.22 U/mg), UP2 (1.19 U/mg), and WTC (0.47 U/mg), respectively. The extracellular protease activity values recorded for the probiotic in the treated composite flour were similar (*p* ≥ 0.05) across 30–60% (v/w) MC.

### Solubilized protein concentration of biotransformed composite flour

**FIGURE 4:**
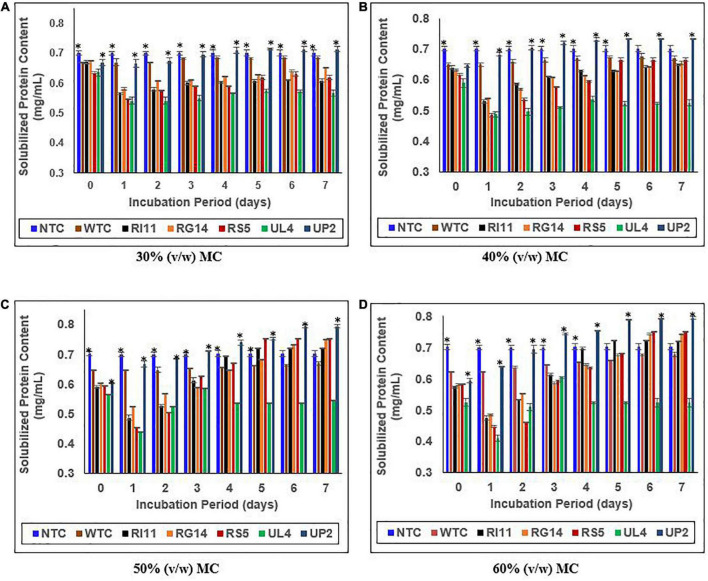
Effect of moisture contents on the soluble protein content of composite flour during 7 days of solid-state fermentation mediated by probiotic strains. Each value represents mean ± SEM, *n* = 3. NTC, non-treated control; WTC, water-treated control; R111, *L. plantarum* RI11; RG14; *L. plantarum* RG14; RS5, *L. plantarum* RS5; UL4, *L. plantarum* UL4; UP2, *P. pentosaceus* UP2. **(A)** 30% (v/w) moisture content, **(B)** 40% (v/w) moisture content, **(C)** 50% (v/w) moisture content, **(D)** 60% (v/w) moisture content. *Indicate significant difference (*p* ≤ 0.05). *^ns^*Not significantly different.

Figure 4 shows the effect of MC (30–60%) (v/w) on the solubilized protein content of the biotransformed composite flour. Surprisingly, on days 0–1, the solubilized protein concentration of probiotics-treated composite flour decreased significantly (*p* ≤ 0.05) across 30–60% (v/w) MC except for the WTC and the UP2-treated composite flour, while the NTC remained stable from days 0 to 7. However, the *L. plantarum* probiotic strains demonstrated a rapid and consistent increase in soluble protein concentration at 50–60% (v/w) MC from days 2 to 7, except for UL4-treated composite flour. The highest percentage of increase (*p* ≤ 0.05) in solubilized protein concentration was observed at 60% (v/w) MC, and UP2-treated composite flour extract showed the highest increase in soluble protein content (33.89%). RS5-treated composite flour recorded 29.31%, which was similar to (*p* ≥ 0.05) RG14 (27.59%) and RI11 (26.34%)-treated composite flour, while WTC recorded a significantly lower percentage of 7.94% from days 0 to 7. The least general increment in soluble protein concentration was recorded at 40% (v/w) MC (UP2, 12.31%; RS5, 8.00%; RI11, 6.25%; RG14, 4.76%; and WTC, 1.54%).

### Crude protein content of biotransformed composite flour

Table 2 presents the crude protein content of the biotransformed composite flour. The MC and probiotic LAB strains demonstrated significant impacts (*p* ≤ 0.05) on the crude protein content of biotransformed composite flour during the SSF process. The WTC recorded a slight increase in protein content (11.99–12.83%) at 60% (v/w) MC from days 0 to 5, whereas the probiotics exerted a significant influence on the protein content of the composite flour at each moisture content during the 7 days of fermentation whereby the highest increase in crude protein content was recorded at 60% (v/w) MC by RG14-treated composite flour (12.10–14.39%), followed by UP2 (12.19–14.28%), RS5 (12.11–14.12%), and RI11 (12.10–13.16%) from days 0–4 of the SSF process. However, the UL4-treated composite recorded a significant decrease in protein content from days 0 to 2, with a gradual increase from days 3 to 7 at 30–60% (v/w) MC. The least increase in crude protein content among the probiotics-treated composite flour was recorded at 30% (v/w) MC.

**TABLE 2:** Effect of moisture contents on the crude protein content (%) of composite flour during solid-state fermentation mediated by probiotic LAB strains for 7 days.

Crude protein content (%)						
Days	WTC	RI11	RG14	RS5	UL4	UP2
**(a) 30% (v/w) moisture content**						
0	11.95 ± 0.03^a–dA^	12.04 ± 0.06^a–dAB^	11.95 ± 0.03^a–dA^	12.08 ± 0.02^a–dA^	11.95 ± 0.01^bA^	12.08 ± 0.06^a–eAB^
1	11.95 ± 0.04^aA^	12.04 ± 0.02^bB^	12.07 ± 0.02^abcABE^	12.41 ± 0.02^cB^	11.96 ± 0.01^abA^	12.01 ± 0.03^abAC^
2	12.05 ± 0.07^bA^	12.21 ± 0.01^bB^	12.24 ± 0.03^bAB^	12.35 ± 0.03^bB–D^	11.48 ± 0.03^bB^	12.20 ± 0.03^bB^
3	12.01 ± 0.03^aA^	12.17 ± 0.02^aB^	12.08 ± 0.02^aBD^	12.37 ± 0.02^b–eBD^	11.53 ± 0.01^ehB^	12.27 ± 0.07^adhA^
4	11.98 ± 0.07^aA^	12.03 ± 0.03^aB^	12.56 ± 0.01^acC^	12.05 ± 0.02^aA^	11.88 ± 0.03^aAB^	12.60 ± 0.01^acD^
5	12.05 ± 0.04^aA^	12.37 ± 0.02^aC^	12.47 ± 0.02^acCD^	12.08 ± 0.02^aAC^	12.04 ± 0.03^aA^	12.04 ± 0.07^acA–C^
6	11.95 ± 0.03^aA^	12.30 ± 0.02^abB^	12.31 ± 0.01^bdD^	12.22 ± 0.04^abAD^	12.03 ± 0.04^adA^	12.08 ± 0.06^abA–C^
7	12.05 ± 0.04^aA^	12.00 ± 0.02^adA^	12.13 ± 0.02^aDE^	12.20 ± 0.08^a–eAD^	11.95 ± 0.02^adA^	12.28 ± 0.07^a–eA–D^
**(b) 40% (v/w) moisture content**						
0	11.99 ± 0.02^a–zA^	12.07 ± 0.04^a–dA^	12.10 ± 0.03^cB^	12.05 ± 0.04^a–dB^	12.05 ± 0.05^a–dB^	12.05 ± 0.04^a–dB^
1	11.98 ± 0.02^abA^	11.99 ± 0.01^abcA^	12.35 ± 0.02^cAC^	12.31 ± 0.01^acBD^	11.90 ± 0.02^abB^	12.44 ± 0.04^cBC^
2	12.02 ± 0.04^bA^	12.21 ± 0.01^bA^	12.24 ± 0.03^bA^	12.94 ± 0.03^acC^	11.31 ± 0.02^bA^	12.87 ± 0.01^bcAD^
3	12.04 ± 0.02^aA^	12.21 ± 0.04^acA^	12.63 ± 0.02^adeA–D^	12.87 ± 0.04^dCE^	11.46 ± 0.02^eAC^	12.93 ± 0.02^dAD^
4	12.01 ± 0.04^abA^	12.81 ± 0.05^bcB^	12.56 ± 0.01^acC^	12.15 ± 0.02^acD^	11.85 ± 0.01^aB^	12.45 ± 0.03^acB^
5	12.09 ± 0.02^abA^	12.81 ± 0.01^bcB^	12.47 ± 0.02^acC^	12.13 ± 0.04^acDE^	12.11 ± 0.06^aBC^	12.04 ± 0.07^acBC^
6	12.01 ± 0.02^abA^	12.30 ± 0.02^bA^	13.19 ± 0.01^cD^	12.25 ± 0.04^adDE^	12.07 ± 0.06^abBC^	12.93 ± 0.03^cD^
7	12.02 ± 0.02^aA^	12.68 ± 0.03^bcAB^	13.13 ± 0.01^eD^	12.20 ± 0.04^acDE^	11.86 ± 0.02^aB^	12.64 ± 0.01^cdC^
**(c) 50% (v/w) moisture content**						
0	12.06 ± 0.06^a–dA^	12.03 ± 0.04^a–dC^	12.00 ± 0.02^a–dA^	12.18 ± 0.02^dA^	12.04 ± 0.02^a–dA^	12.12 ± 0.04^a–dA^
1	12.01 ± 0.02^abA^	12.43 ± 0.04^cA^	12.69 ± 0.06^cdAB^	12.73 ± 0.01^cB^	11.75 ± 0.01^aB^	13.00 ± 0.02^cdB^
2	12.11 ± 0.06^bA^	12.68 ± 0.06^abBC^	12.93 ± 0.01^acB^	13.03 ± 0.01^adC^	10.84 ± 0.01^eC^	13.46 ± 0.04^dB^
3	12.21 ± 0.04^abA^	12.81 ± 0.01^dB^	13.51 ± 0.06^dfgC^	13.44 ± 0.01^efD^	11.17 ± 0.01^eD^	14.13 ± 0.02^eC^
4	12.77 ± 0.06^cB^	13.06 ± 0.02^cBC^	14.25 ± 0.01^dD^	14.03 ± 0.05^fE^	11.90 ± 0.03^aAB^	14.16 ± 0.01^fC^
5	12.81 ± 0.05^cB^	13.05 ± 0.01^cC^	14.24 ± 0.01^fD^	14.05 ± 0.02^fE^	11.83 ± 0.05^aAB^	14.17 ± 0.02^fC^
6	12.82 ± 0.04^cB^	13.04 ± 0.02^cC^	14.25 ± 0.06^eD^	13.99 ± 0.06^eDE^	11.85 ± 0.05^abAB^	14.05 ± 0.02^fC^
7	12.79 ± 0.01^bB^	13.04 ± 0.02^eC^	14.23 ± 0.01^fD^	14.03 ± 0.02^gE^	11.85 ± 0.01^adE^	14.12 ± 0.01^ghC^
**(d) 60% (v/w) moisture content**						
0	11.99 ± 0.05^a–dA^	12.10 ± 0.07^a–dA^	12.04 ± 0.05^a–dA^	12.11 ± 0.07^a–dA^	11.94 ± 0.02^bC^	12.19 ± 0.01^a–dA^
1	11.97 ± 0.01^abA^	12.38 ± 0.01^cB^	12.83 ± 0.01^cdB^	12.70 ± 0.04^cAB^	10.74 ± 0.05^eAB^	12.97 ± 0.02^dB^
2	12.14 ± 0.03^bA^	13.16 ± 0.02^acCE^	13.40 ± 0.01^dC^	12.99 ± 0.01^adB^	10.81 ± 0.01^eB^	13.49 ± 0.04^dC^
3	12.14 ± 0.02^abA^	12.85 ± 0.03^dCD^	14.26 ± 0.02^fD^	14.12 ± 0.02^gC^	11.12 ± 0.01^eC^	14.28 ± 0.02^fD^
4	12.81 ± 0.07^cB^	12.99 ± 0.01^cCDE^	14.39 ± 0.01^dD^	14.05 ± 0.04^fC^	11.85 ± 0.03^aD^	14.19 ± 0.03^dfD^
5	12.83 ± 0.04^cB^	13.15 ± 0.01^cCDE^	14.38 ± 0.01^dD^	14.01 ± 0.07^fC^	11.86 ± 0.03^aD^	14.17 ± 0.03^dfD^
6	12.83 ± 0.03^cB^	12.95 ± 0.03^cDE^	14.37 ± 0.01^eD^	14.01 ± 0.04^efC^	11.83 ± 0.03^aD^	14.13 ± 0.04^efD^
7	12.45 ± 0.03^cB^	13.08 ± 0.07^eDE^	14.37 ± 0.02^fD^	13.84 ± 0.07^efgC^	11.88 ± 0.01^aD^	14.15 ± 0.02^fhD^

Values are mean ± standard error of the mean (SEM). Mean values in the same column having the same superscript (a–h) are not significantly different at *p* ≤ 0.05. Mean values in the same row having the same superscript (A–E) are not significantly different at *p* ≤ 0.05. NTC, non-treated control; WTC, water-treated control; R111, *L. plantarum* RI11; RG14, *L. plantarum* RG14; RS5, *L. plantarum* RS5; UL4, *L. plantarum* UL4; UP2, *P. pentosaceus* UP2. (a) 30% (v/w) moisture content, (b) 40% (v/w) moisture content, (c) 50% (v/w) moisture content, and (d) 60% (v/w) moisture content. The protein content value for NTC was between 11.98 and 12.13%.

### *In vitro* protein digestibility of biotransformed composite flour

**FIGURE 5:**
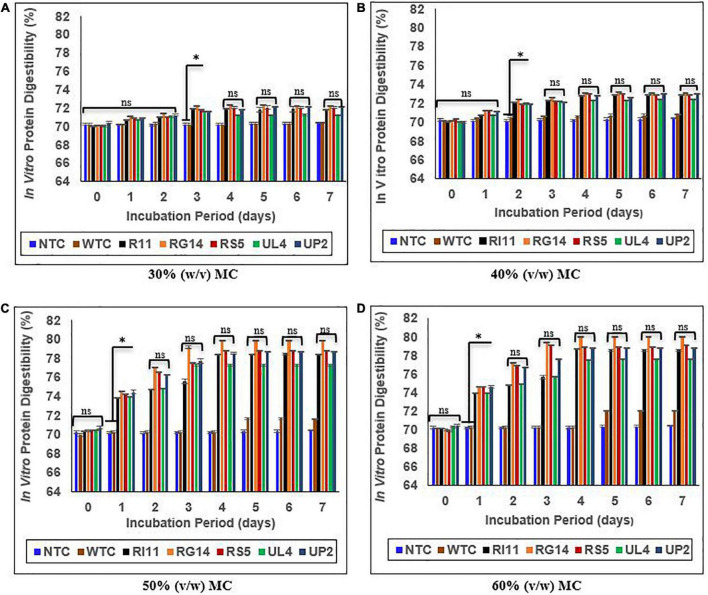
Effect of moisture contents on *in vitro* protein digestibility of composite flour during 7 days of solid-state fermentation mediated by probiotic strains. Each value represents mean ± SEM, *n* = 3. NTC, non-treated control; WTC, water-treated control; R111, *L. plantarum* RI11; RG14; *L. plantarum* RG14; RS5, *L. plantarum* RS5; UL4, *L. plantarum* UL4; UP2, *P. pentosaceus* UP2. **(A)** 30% (v/w) moisture content, **(B)** 40% (v/w) moisture content, **(C)** 50% (v/w) moisture content, **(D)** 60% (v/w) moisture content. *Indicate significant difference (*p* ≤ 0.05). *^ns^*Not significantly different.

Figure 5 presents the *in vitro* protein digestibility (IVPD) of biotransformed composite flour at 30–60% (v/w) MC of SSF. The IVPD for NTC was between 70.17 and 70.41%. A rapid increase in IVPD was observed for the probiotics-treated composite flour at 50 and 60% (v/w) MC from days 0 to 4, although the highest percentage increase (*p* ≤ 0.05) was recorded at 60% (v/w) MC. RG14-treated composite flour recorded the highest increase of 14.26%, followed by (*p* ≥ 0.05) RS5-treated composite flour, 13.17%; RI11-treated composite flour, 12.30%; UP2-treated composite flour, 11.91%; UL4-treated composite flour, 10.44%; and WTC displayed the lowest percentage of 2.69%. The least percentage increase in IVPD was observed at 30% (v/w) MC, in which RG14-treated composite flour recorded 3.10% of IVPD, followed by RS5 (2.71%), RI11 (2.69%), UP2 (2.44%), and UL4 (2.14%).

### Evaluation of the overall performances of the probiotics using a competitive analysis tool

**FIGURE 6:**
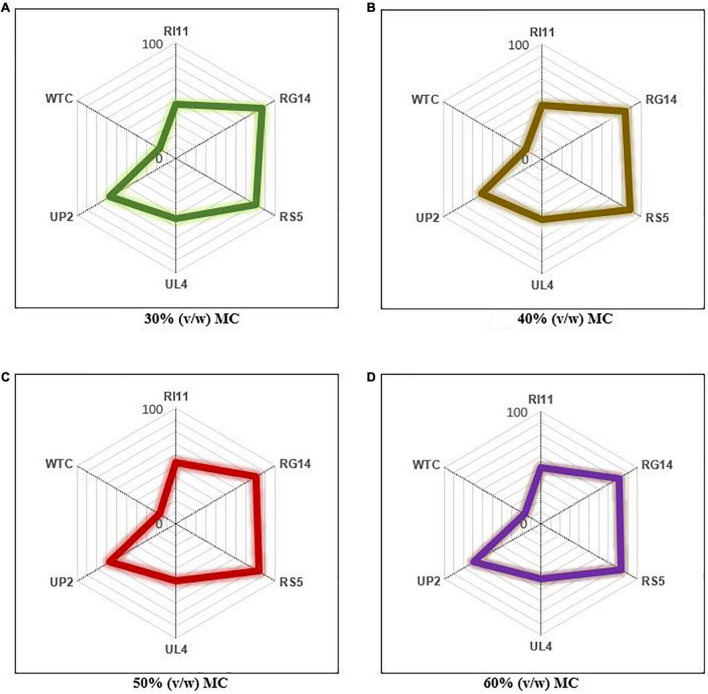
Ranking of the overall performance of probiotics at 30–60% (v/w) moisture contents for the biotransformation of composite flour via solid-state fermentation for 7 days. WTC, water- treated control; R111, *L. plantarum* RI11; RG14, *L. plantarum* RG14; RS5, *L. plantarum* RS5; UL4, *L. plantarum* UL4; UP2, *P. pentosaceus* UP2. **(A)** 30% (v/w) moisture content, **(B)** 40% (v/w) moisture content, **(C)** 50% (v/w) moisture content, **(D)** 60% (v/w) moisture content. The percentage score of each strain represents their performance. The strain with the highest score at each moisture content is considered the best probiotic strain for the biotransformation of composite flour (Microsoft excel spreadsheet version 2010).

The overall performance of studied probiotics was evaluated at each moisture content for all the parameters analyzed using a competitive analysis tool (Microsoft Excel Spreadsheet Version 2010). A score of 1–6 was assigned to the results obtained from WTC and probiotics-treated composite flours and expressed on a full scale of 100, as shown in Figure 6. The overall highest improvement in the analyzed parameters was obtained at 60% (v/w) MC, and RS5-treated composite flours showed the best performance with the highest score of 83.35%, followed by RG14 (80.57%), UP2 (69.46%), UL4 (50.01%), RI11 (50.01%), and WTC (16.67%). The trend of the scores obtained at 60% (v/w) MC was similar to the scores obtained at 50% (v/w) MC. The least improvement in composite flour properties was recorded at 30% (v/w) MC, and RG14-treated composite flour had the highest performance score of 86.13%, followed by RS5 (80.57%), UP2 (66.68%), UL4 (52.79%), RI11 (47.23%), and WTC (16.67%).

### Regression analysis of the analyzed variables at different moisture content as a function of biotransformation period

Table 3 presents the regression models for the interaction between the moisture contents and biotransformation duration of the analyzed parameters (pH, %TTA, extracellular protease activity, soluble protein, crude protein, and *in vitro* protein digestibility). No significant interaction (*p* ≥ 0.05) was noted between the MC and biotransformation duration for the viable cell count of LAB, whereas a significant interaction (*p* ≤ 0.05) was observed for the pH, %TTA, extracellular protease activity, soluble protein, crude protein and *in vitro* protein digestibility of the biotransformed composite flour.

**TABLE 3:** Regression equations of analyzed variables at different moisture contents as a function of the fermentation period.

Variables	Moisture contents	Equations	*R* ^2^
LAB count (LOG CFU/g)	30% (v/w)	*y* = 6.68−0.01 X^ns^	0.02
	40% (v/w)	*y* = 7.03−0.01 X^ns^	0.01
	50% (v/w)	*y* = 7.43−0.04 X^ns^	0.02
	60% (v/w)	*y* = 7.52−0.01 X ^ns^	0.01
pH	30% (v/w)	*y* = 5.64−0.17 X*	0.56
	40% (v/w)	*y* = 5.57−0.17 X*	0.54
	50% (v/w)	*y* = 5.52−0.18 X*	0.52
	60% (v/w)	*y* = 5.50−0.18 X*	0.51
%TTA	30% (v/w)	*y* = 0.51 + 0.09 X***	0.77
	40% (v/w)	*y* = 0.59 + 0.09 X***	0.71
	50% (v/w)	*y* = 0.64 + 0.09 X**	0.65
	60% (v/w)	*y* = 0.66 + 0.09 X**	0.64
Specific protease activity (U/mg)	30% (v/w)	*y* = 0.91 + 0.04 X*	0.5
	40% (v/w)	*y* = 1.34 + 0.04 X*	0.55
	50% (v/w)	*y* = 2.23 + 0.11 X**	0.63
	60% (v/w)	*y* = 2.26 + 0.12 X**	0.66
Soluble protein content (mg/mL)	30% (v/w)	*y* = 0.63 + 0.02 X^ns^	0.09
	40% (v/w)	*y* = 0.61 + 0.01 X*	0.5
	50% (v/w)	*y* = 0.58 + 0.02 X**	0.81
	60% (v/w)	*y* = 0.56 + 0.02 X**	0.83
Crude protein content (%)	30% (v/w)	*y* = 12.05 + 0.02 X*	0.5
	40% (v/w)	*y* = 12.12 + 0.05 X***	0.73
	50% (v/w)	*y* = 12.25 + 0.20 X***	0.8
	60% (v/w)	*y* = 12.23 + 0.20 X**	0.86
*In vitro* protein digestibility (%)	30% (v/w)	*y* = 70.49 + 0.18 X**	0.79
	40% (v/w)	*y* = 70.64 + 0.28 X**	0.81
	50% (v/w)	*y* = 12.25 + 0.20 X***	0.8
	60% (v/w)	*y* = 71.99 + 0.83 X**	0.79

^*ns*^Not significant, significant. *p ≤ 0.05. **p ≤ 0.01. ***p ≤ 0.001. *n* = 3.

## Discussion

Composite flour has a wide array of applications in the food industry, and the flour composition was selected mainly to improve the nutritional and functional properties of composite food products (9, 35). The high consumption of rice, sorghum, and soybean in Asia and Africa and the consumers’ preference level for soybean in composite food products (4, 5, 9–11, 35) were considered for the formulation of composite flour, whereby the ratio of 45:45:10 for rice, sorghum, and soybean was selected in the current study. In addition, the MC was optimized to determine the minimum moisture that could promote overall improvement in the composite flour’s physicochemical properties.

### Cell viability of probiotics

Microorganisms require water to support their growth generally (15). MC is an important factor affecting flour quality produced *via* the SSF process (15, 26, 27). The tested probiotics demonstrated the capability to grow well at 30–60% (v/w) MC. Nevertheless, LAB growth was not detected in the raw composite flour (NTC) using MRS selective medium. Other groups of bacteria could be present in the raw flour since we did not determine their growth using the nutrient medium. The microorganisms in fermented food could originate from the raw materials and process equipment (41), and the detection of LAB in the water-treated (WTC) composite flour may have been introduced during the preparation of the SSF process. However, the LAB recorded in the WTC was significantly lower than those reported by Pranoto et al. (25) and Ogodo et al. (26, 27) during submerged fermentation of sorghum and soybean flour. The rapid growth of selected probiotics strains share a similar trend with the LAB growth reported for rice flour fermentation (42–44) and subsequently maintained growth stability, which was in line with the findings of Giri et al. (45) during the preparation of rice-based fermented beverage with *L. plantarum* L7. Meanwhile, Ogodo et al. (26, 27) reported a decrease in the LAB population at 48 h during sorghum and soybean fermentation. The MC of 50 and 60% was observed to have comparable effects on the probiotic populations and was similar to the MC reported for the SSF of cereal, and legume flours mediated by LAB (46–49). Moreover, Rodriguez de Olmos et al. (20, 50) demonstrated that *Lactobacillus* could grow well at 50–55% (v/w) MC during the optimization of soy SSF parameters and reported a similar increase in the LAB population at 60 and 80% (v/w) MC. In the present study, *Enterobacteriaceae* growth was not detected on EMB agar during the 7 days of fermentation for both the NTC, WTC, and probiotic-treated composite flours. Gram-negative bacteria usually require relatively higher water activity than Gram-positive bacteria (51). Hence, the absence of *Enterobacteriaceae* count could be attributed to the limited water activity of SSF. In addition, the probiotic LAB strains employed in this study have been reported for their broad antimicrobial activities (15, 30, 52).

### pH and total titratable acidity of biotransformed composite flour

Microorganisms such as LAB produce organic acids (lactic and acetic acid) and other metabolites during the fermentation process, which causes a reduction in pH and subsequent improvement in the organoleptic properties and safety quality of the final products (53). The selected probiotics strains produced a substantial amount of organic acids, which caused a significant reduction in pH of the biotransformed composite flour in a trend similar to those reported by Rodriguez de Olmos et al. (20) at 24 h of soybean SSF mediated by LAB. A slight reduction in the pH observed in the WTC could be due to the presence of LAB and other organic acid-producing bacteria in the composite flour, although the result was lower than those reported for rice and sorghum flour that naturally fermented *via* submerged conditions (27, 54). Furthermore, microorganisms vary in their capability of organic acid production particularly lactic acid (55), and the selected probiotic strains sufficiently demonstrated their capacity during the SSF process, whereby the RS5 strain caused the highest acidification at 30–60% (v/w) MC in the treated composite flour. These findings were consistent with the recent report on the characterization of organic acids composition produced by similar probiotic *Lactiplantibacillus plantarum* strains (52). SSF promotes a higher concentration of product yield (56); thus, the %TTA obtained in RS5, and UL4-treated composite flour at 40–60% (v/w) MC was higher than the sorghum flour treated with *L. plantarum* NBRC 15891 strain *via* submerged fermentation (25).

### Specific endoprotease activity of biotransformed composite flour extract

The recent application of the SSF process was focused on the production of hydrolytic enzymes (57, 58). Proteases are vital industrial enzymes with numerous applications in the food and feed processing, detergent, and pharmaceutical industries (15). The presence of protease enzymes in gluten-free flour enhances the baking and sensory quality of bread and promotes the overall functionality and digestibility of food protein (59, 60). The production of protease enzymes by various microorganisms under SSF conditions has been documented (15, 57). Probiotic LAB is known to have a well-established proteinase/peptidase system to catalyze the degradation of complex proteins into shorter peptides and free amino acids (28, 61). Recently, the extracellular secretion of protease enzymes by the selected probiotic LAB was demonstrated using an MRS medium and subsequently applied for PKC fermentation (16, 28, 29). Interestingly, the observation in this study was consistent with the previous reports (28, 29). Finally, the increase in specific endoprotease activity observed in the WTC could be due to the activation of endogenous protease present in composite flour or from the activity of the indigenous bacteria (62).

### Soluble protein content of biotransformed composite flour

Protein solubility is an important functional feature in the development of new protein ingredients (63). Soluble protein includes the mixture of enzymes, free amino acids, and small soluble peptides (bioactive peptides) present within the fermentation media. Naturally, microorganisms require carbon, nitrogen, phosphorus, and other minerals to sustain growth and to carry out biochemical processes (64). Probiotic LAB are fastidious microorganisms that have been widely reported to exhibit high amino-acid requirements for their growth (55), and this was demonstrated on days 0–1 in the probiotic *L. plantarum* treated composite flour. Subsequently, the active secretion of endoprotease enzyme and biosynthesis of amino acids by the probiotic LAB would have contributed greatly to the rapid increase in soluble protein concentration of the treated composite (16) except for *L. plantarum* UL4 that was reported to have an exigent requirement for amino acids (29). Rodriguez de Olmos et al. (50) reported a decrease in amino acids at 24 h of LAB fermented soybean, but an increase in amino acids at the end of the SSF process was recorded. Similar findings by Lee et al. (16) revealed a lower and steady increase in soluble protein when the probiotic *L. plantarum* strains were applied to PKC under SSF conditions. Contrary to the high amino-acid requirement exhibited by probiotic *L. plantarum* strains on days 0–1, *Pediococcus pentosaceus* UP2 demonstrated a limited amino-acid requirement. *Pediococcus* sp. has been demonstrated to have a robust amino-peptidase activity compared to *Lactobacillus* sp.; hence, they can secrete higher amino acids (28, 65). Surprisingly, the percentage increase in soluble protein recorded for the treated composite flour was in the range observed for soluble free amino nitrogen reported for the treatment of oats flour by *L. plantarum* TK9 and *Bif. animalis* subsp. *lactis* V9 *via* SSF (46). Lysine is one of the major limiting amino acids in cereal foods, and bacteria fermentation was reported to boost lysine content in cereal protein (8, 62). Hence, an appreciable amount of lysine could be present in the UP2-treated composite flour because the UP2 strain was recently reported to exhibit high production of lysine amino acid (29), while soybean is a legume that is rich in lysine amino acid (8).

### Protein content of biotransformed composite flour

Protein is a major component of the human diet, and it serves as the building block for vital organs, muscles, hormones, and blood (13). The insufficient intake of adequate-quality protein often results in PEM symptoms (66). The protein content of raw composite flour or mixture depends on the variety of individual grain components used and the geographical location of planting (67, 68). The protein content of NTC was in a similar range to those reported for composite flour derived from malted sorghum and soybean flour (69), malted sorghum, sprouted soybean and carrot flour (70), and rice, sorghum, and bamboo shoot (71). The significant increase in protein in this study could be due to the high extracellular protease activity corresponding to the probiotic LAB viability. Reports have suggested that the secretion of protease enzymes and an increase in bacteria population or biomass during the fermentation process contributed to an increase in protein content (26, 27). The range of the population of the selected probiotic strains in the treated composite flour was 4–7 log_10_ CFU/g higher than the WTC. In addition, Osman et al. (72) have attributed the reduction in carbohydrates (loss of dry matter) during the fermentation process to the increment in protein content. However, a decrease in protein content was observed in the UL4-treated composite flour. The high protease enzyme activity and cell population suggested that the UL4 would have utilized the protein of the composite flour for its growth and other related metabolism activities. Nor et al. (73) noted UL4 was the highest producer of folate (vitamin B9), while Moghadam et al. (74) also observed the simultaneous presence of two bacteriocin-producing structural genes (*plantaricin* EF and *plantaricin* W) in UL4 strain. PEM is prevalent in Africa and Asia, accounting for nearly 45% of mortality among children under the age of five (75, 76). According to the WHO/FAO/UNU Report (77) on estimated average requirement (EAR) and recommended daily allowance (RDA) for protein requirement in human nutrition, the results of crude protein obtained for RG14-, UP2-, and RS5-treated composite flour meet the adequate level of satisfaction (LOS) for male and female children under 6 years of age (66). The protein contents of RG14-, UP2-, and RS5-treated composite flour were approximately 14.00 g/100 g of composite flour, while the reference EAR/RDA for male and female children <6 years of age were 11.30/13.70 g/day and 10.70/13.00 g/day (66, 77), respectively. LOS was calculated as the protein content of composite flour (14.00 g) divided by the EAR/RDA multiplied by 100 (66). LOS greater than 100% EAR/RDA is considered to be adequate (66). Therefore, the estimated LOS for food prepared with 100 g of RG14-, UP2-, and RS5-treated composite flour for male and female children <6 years of age were 123.89% EAR/102.19% RDA and 130.84% EAR/107.69% RDA, respectively.

### *In vitro* protein digestibility of biotransformed composite flour

The improvement in protein digestibility of food products indicates the availability of protein for body absorption or utilization (78). Thus, crude protein digestibility is an important component of protein quality (77, 79, 80). To the best of our knowledge, there is no report on the effect of SSF mediated by LAB on the protein digestibility of composite flour. According to Rodriguez de Olmos et al. (50), the applications of LAB in the fermentation process to improve the protein digestibility of food were reported for submerged fermentation (SMF). However, this study was focused on the protein digestibility of probiotics-treated composite flour (Figure 5) under the SSF system. A significant increase in protein digestibility was noted for the probiotics-treated composite flour, especially at 50 and 60% (v/w) MC, attributed to the active secretion of extracellular protease enzyme by the selected probiotics. This observation was in agreement with several fermentation studies that have demonstrated the secretion of hydrolytic enzymes and low pH conditions that would favor the peptidase activity to improve protein digestibility (25–27, 78). Exogenous supplementation of protease enzymes in feed ingredients or food by fermenting bacteria can reduce protease and trypsin inhibitors that block the activity of digestive protein enzymes (12, 80). Therefore, the extracellular proteolytic enzymes reported in this study could have reduced the protease and trypsin inhibitors drastically to cause a significant change in protein digestibility. SSF process offers a simple technique; it could be applied to agro-waste with a limited risk of contamination compared to SMF (81, 82), although the percentage increase in IVPD of fermented composite flour as obtained in this study was much lower than those reported for SMF of sorghum flour (25, 27). A similar and lower percentage of IVPD has been reported for SSF of soybean and lupine flour (83) and soybean seeds (24) by LAB, respectively.

### Interaction between the moisture contents and biotransformation period (days)

The relationship between the MC and biotransformation period for the analyzed parameters was considered in the study. A significant interaction between 30 and 60% (v/w) MC and the biotransformation period was observed for the pH of the biotransformed composite flour (*R*^2^ = 0.51–0.56, *p* ≤ 0.05), followed by the %TTA (*R*^2^ = 0.64–0.77, *p* ≤ 0.05), specific protease activity (*R*^2^ = 0.50–0.66, *p* ≥ 0.05), *in vitro* protein digestibility (*R*^2^ = 0.79–0.81, *p* ≤ 0.05) and crude protein content (*R*^2^ = 0.50–0.86, *p* ≤ 0.05). The interaction between MC and biotransformation period for the soluble protein content was not significant at 30% (v/w) MC (*R*^2^ = 0.09, *p* ≥ 0.05), while 40–60% (v/w) MC was significant (*R*^2^ = 0.50–0.83, *p* ≤ 0.05). No significant interaction was recorded for LAB count (*R*^2^ = 0.01–0.02, *p* ≥ 0.05) regardless of the MC. Finally, a similar trend of interaction between MC and the biotransformation period was observed for all the analyzed variables; however, 50% (v/w) MC was considered the best due to the significant improvement recorded in the composite flour properties and the preference for flour quality.

### Overall probiotic biotransformation performance on composite flour

The physical parameters (pH, %TTA) and protein quality-related features (specific endoprotease activity, soluble protein, crude protein, and IVPD) of the treated composite flour were considered for the overall probiotic biotransformation performance analysis. The ranking results illustrated that RS5 was the best probiotic strain for the biotransformation process at 40–60% (v/w) MC, even though the highest overall improvement or increase in value of analyzed parameters was noted at 60% (v/w) MC. RS5 strain recorded the highest reduction in pH and increase in %TTA among the probiotics-treated composite flour, and these attributes could be employed as indicators of fermentation performance, which is one of the major selection criteria for fermentation starter cultures (26, 27, 84, 85). Moreover, LAB are Gram-positive non-spore-forming bacteria that are less sensitive to reduced water activity (51). Thus, probiotic LAB strains employed in this study exhibited comparable responses and performance in SSF at 50 and 60% (v/w) MC.

## Conclusion

Composite flour derived from rice, sorghum, and soybean could play a significant role in addressing the burden of PEM in developing countries. Therefore, the improvement of the nutritional quality of composite flour is vital. This study has demonstrated the capability of the selected probiotic LAB to grow on composite flour and share similar traits, such as the production of organic acids and secretion of extracellular protease enzymes to cause improvement in the quality of composite flour under SSF conditions. However, the potential to produce organic acids and secrete proteolytic enzymes was strain dependent. RS5-treated composite flour exhibited the highest organic acid production and pH reduction, RG14-treated composite flour showed the highest endoprotease activity and improvement in crude protein and protein digestibility, while UP2-treated composite flour recorded the highest increase in soluble protein concentration. Overall, the RS5 strain exhibited the best performance for the SSF fermentation of rice, sorghum, and soybean composite flour. In addition, the ability of the selected probiotic LAB to thrive and transform the composite flour at 50 and 60% (v/w) MC offers an excellent advantage for commercial application. Lower moisture content will promote flour quality; thus, 50% (v/w) MC was considered to be the most suitable MC for the effective biotransformation of gluten-free composite flour of rice, sorghum, and soybean. Therefore, applying the selected probiotics as a biotransformation agent warrants further investigations into their impact on other essential nutritional components, functional properties, and health-promoting features of the composite flour.

## Data availability statement

The original contributions presented in this study are included in the article/supplementary material, further inquiries can be directed to the corresponding author.

## Author contributions

HF and TL: conceptualization, funding acquisition, project administration, and resources. HF, NR, and TL: data curation, supervision, and validation. KK and HF: formal analysis, investigation, and methodology. KK, NR, and HF: writing—original draft. KK, HF, and NR: writing—review and editing. All authors have read and agreed to the published version of the manuscript.
